# The missing story behind Genome Wide Association Studies: single nucleotide polymorphisms in gene deserts have a story to tell

**DOI:** 10.3389/fgene.2014.00039

**Published:** 2014-02-18

**Authors:** William Schierding, Wayne S. Cutfield, Justin M. O'Sullivan

**Affiliations:** ^1^Liggins Institute, University of AucklandAuckland, New Zealand; ^2^Gravida – National Centre for Growth and DevelopmentAuckland, New Zealand

**Keywords:** GWAS, epigenetics, DNA folding, chromosomal folding, gene deserts

## Abstract

Genome wide association studies are central to the evolution of personalized medicine. However, the propensity for single nucleotide polymorphisms (SNPs) to fall outside of genes means that understanding how these polymorphisms alter cellular function requires an expanded view of human genetics. Integrating the study of genome structure (chromosome conformation capture) into its function opens up new avenues of exploration. Changes in the epigenome associated with SNPs in gene deserts will allow us to define complex diseases in a much clearer manner, and usher in a new era of disease pathway exploration.

## INTRODUCTION

Studies of human variation and its links to disease promise to usher in an era of personalized medicine, where the genetic code of an individual is assessed and used to guide clinical practice. This advance toward individualized medicine is being made possible through the cataloging of common genetic variants and their associations with complex traits and disease.

**FIGURE 1:**
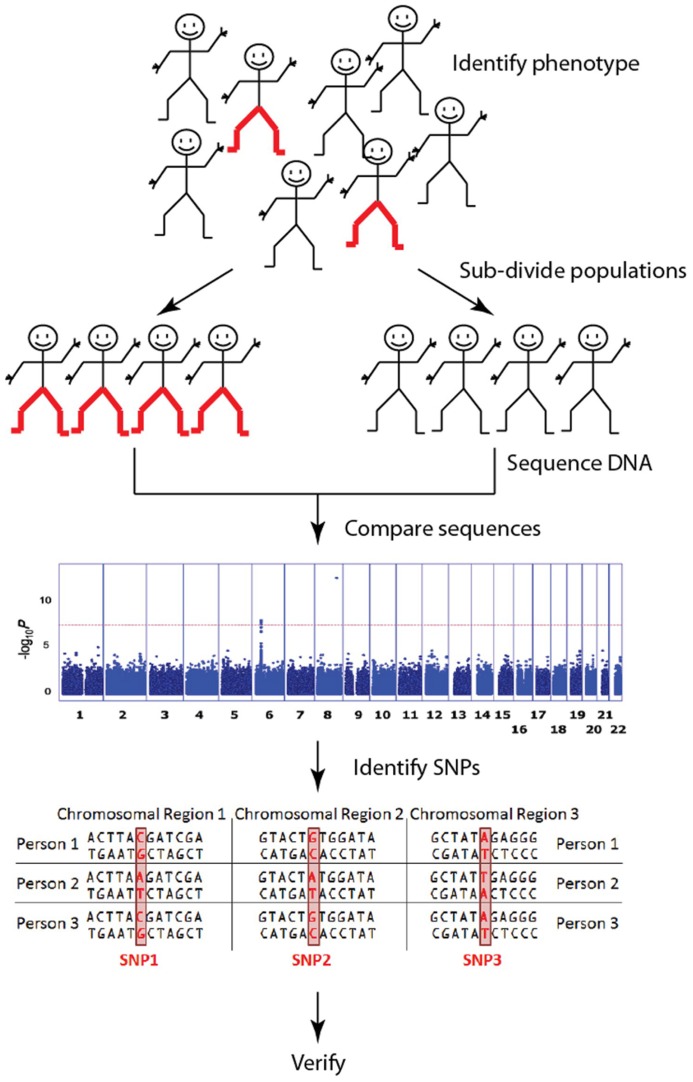
**Genome wide association studies (GWAS) identify genetic associations by comparing common single nucleotide polymorphisms (SNP; a single DNA base which changes in the genome between paired human chromosomes) across the human genome within a case cohort (red-pants) with those present in a control cohort (black-pants).** The result is an “unbiased” discovery of common genetic variants associating with a particular trait/disease. These are typically presented in a Manhattan plot. [The Manhattan plot shown illustrates the significance of the trait association (y-axis) according to genome position (x-axis) for pediatric asthma (Noguchi et al., 2011). In this study, a SNP in the 8q24 gene desert was clearly identified as the most significant risk loci for pediatric asthma]. There are millions of SNPs across the billions of base pairs of DNA that make up the human genome, but current GWAS focus mostly on the relatively few SNPs that fall within genes. Although each SNP has a relatively small impact on any one trait/disease, together they explain large amounts of variation.

Genome wide association studies (GWAS) have a central role in this human genetics revolution. The primary purpose of GWAS is to identify single nucleotide polymorphisms (SNPs) that are associated with phenotypic traits, typically those associated with a particular disease (**Figure 1**). In a handful of cases, particularly in cancer, the move to genome wide SNP detection has led to clinically relevant predictions (Jostins and Barrett, 2011). However, despite these best-case-scenarios, nearly half of the disease-associated SNPs from published GWAS are not located in or near genes (Visel et al., 2009; Hindorff et al., 2013). Therefore, despite the fact that significant associations are often found between complex traits and SNPs in gene deserts (i.e., genomic regions of > 500kb that lack annotated genes or protein-coding sequences; Venter et al., 2001; Libioulle et al., 2007; Grant et al., 2009), their location within gene deserts means they have no readily annotated gene function and cannot be assigned to a specific biological pathway. Explanations abound for why apparently significant SNPs are located in gene deserts (Visel et al., 2009; Uddin et al., 2011; Zhang et al., 2012). However, typically these sorts of results only make their way into publications as part of supplementary tables of findings, often without any attempt to explain the association. Current trends are now shifting to focus on SNPs within gene deserts as potentially contributing to diseases by regulating gene function from a distance.

How is it possible for SNPs within gene deserts to regulate unlinked genes? The answer lies within the twists and turns that form when 3 m of human DNA (chromosomes) is packaged within a roughly spherical nucleus that is only approximately 10 μm in diameter. Within the hierarchy of folding necessary to package the genome within the eukaryotic nucleus, regions of each chromosome contact other chromosomes to form an intricate 3-dimensional DNA network. Therefore, while two regions of DNA (loci) may be distant on a linear scale, DNA folding provides a mechanism for these two loci to become spatially close together. Implicit in this concept is the idea that all genetic functions (regulation, reading, repair, and replication) are influenced by this 3-dimensional architecture, generating the cell’s morphology and function (Misteli, 2001). Intra-cellular DNA structure cannot be divorced from its functions.

**FIGURE 2:**
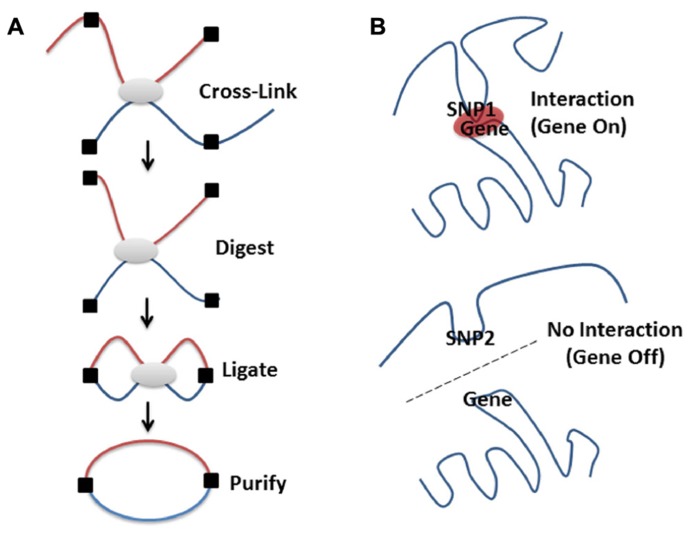
**High-resolution molecular techniques for studying the spatial organization of chromosomes.** Various methods exist to capture when two genomic regions are in close spatial proximity. Microscopy is a powerful tool for visualizing structure, especially when combined with FISH methodologies (Branco and Pombo, 2006; Rouquette et al., 2010). However, even the super resolution microscopes only resolve 15–20 nm structures, which limits the ability to visualize the lowest levels of DNA packing (Baker, 2011). By contrast, proximity based ligation technologies [e.g., 3C (Dekker et al., 2002), 4C (Zhao et al., 2006), GCC (Rodley et al., 2009), ChIA-PET (Li et al., 2013 n.d.), or 5C (Dostie et al., 2006)] enable the high-resolution identification of which DNA sequences are contacting each other, directly or indirectly, in 3D space at a given moment in time. **(A)** Proximity based ligation methodologies currently incorporate the same basic steps. The structure of the chromatin is captured by cross-linking which ensures that regions that are in contact are chemically held together. The chromatin structure is then broken into pieces with restriction enzymes (digested), pieces that are held together by cross-links are then enzymatically joined together (ligated), purified away from the cellular debris and detected by various methods (for a more in depth discussion see Grand et al., 2011). The method used for detection of these ligated products (i.e., PCR, low or high through-put sequencing) depends on whether specific, close range or global organization is being investigated. By coupling 3C technologies to next-generation sequencing (NGS) it is possible to create unbiased low or high-resolution 3-dimensional maps of whole genomes (Lieberman-Aiden et al., 2009; Rodley et al., 2009). This has led to discoveries of interesting interactions in regions of the DNA that otherwise wouldn’t be thought to be part of a particular disease process (Rodley et al., 2012). **(B)** Genetic variation (SNPs, see **Figure 1A**) can affect chromatin structure by altering folding patterns. These changes result in differential spatial relationships, leading to a gain or loss of function through altered associations between enhancers, promoters, LCRs, silencers, or imprinting control regions.

Methods to study intra-cellular DNA organization, e.g., chromosomal conformation capture, 3C, or related methodologies (**Figure 2**) have confirmed that loci on separate chromosomes (Ling et al., 2006; Lomvardas et al., 2006) or on the same chromosome but separated by large intervening sequences (Carter et al., 2002; Tolhuis et al., 2002; Sotelo et al., 2010; French et al., 2013) can interact in space to regulate the expression of multiple genes (Schoenfelder et al., 2010). Therefore, it is possible that intergenic SNPs associated with diseases are indeed involved in the regulation of genes and pathways through spatial associations with different genes. In effect, intergenic SNPs represent sequence alterations that affect the ability of these regions to interact with other loci or recruit the proteins necessary for gene regulation at a distance (Sotelo et al., 2010; French et al., 2013). This proposal gains some support from the finding that 76% of GWAS SNPs are near (or in linkage within a haplotype block) DNaseI hypersensitive sites, which are often locus control regions (LCRs) – regions associated with enhancers (Maurano et al., 2012; Malin et al., 2013). Despite the fact that “our understanding of higher-order genomic structure is coarse, fragmented and incomplete,” (Dixon et al., 2012) there are certain things that have been learned by a decade of research on the topic.

## HOW DO LONG-DISTANCE INTERACTIONS AFFECT GENE EXPRESSION?

The “dog-on-a-lead” model has been proposed to account for the coordination of positioning of chromosomes and the formation of interactions within and between chromosomes. In this model, chromosome positioning and folding dominates the 3-dimensional organization of the nucleus, while genes, domains, and enhancers are largely limited to form contacts within the chromosomal context within which they are located (Krijger and de Laat, 2013). This model allows for the occurrence of cell-specific interactions within similar chromosome domains as a cause of variegated gene expression amongst otherwise identical cells. (de Wit et al., 2013).

The dog-on-a-lead model for the spatial organization of eukaryotic genomes encompasses the formation of short and long-distance interactions, between enhancers and promoters, which form an essential component of the regulatory systems for eukaryotic gene expression (Sotelo et al., 2010; French et al., 2013). These interactions are hypothesized to function to bring genes and regulatory regions to spatial domains which contain high concentrations of the relevant enzymes, proteins and raw materials required for the production of messenger RNA. In effect, loci co-localize at factories that promote transcription (Cook, 2002). For example, activation of the HoxB and uPA genes is accompanied by alterations in their spatial organization that include the association of these genes into a factory that promotes their transcription (Chambeyron and Bickmore, 2004; Marenduzzo et al., 2007; Ferrai et al., 2010). Thus, inter- and intra-chromosomal organization is a reflection of the cell machinery integrating spatial relationships into higher-order epigenomic regulation (Baker, 2011).

However, as clear as these models have become, technological challenges have left questions about the existence, formation and maintenance of transcription factories that require chromosome-mixing and long-range interactions. Firstly, the 3C based methods that have become popular for the study of the 3-dimensional chromosome networks can potentially bias the results (Razin et al., 2010; Gavrilov et al., 2013). Moreover, these methods are probabilistic (O’Sullivan et al., 2013) and thus, interactions identified by 3C are representative of what is happening in a population of cells. Any one cell in the population is likely to display only a small proportion of the interactions that were captured. Therefore, the “factory” may not actually be present in any one cell. Secondly, the amount of mixing (Branco and Pombo, 2006) that occurs between spatially adjacent chromosomes within the nucleus remains controversial with some contending that chromosomes are self-contained with little mixing (Olivares-Chauvet et al., 2011).

## IS GENOME ORGANIZATION STABLE?

Evidence exists for organization of chromosomes into topological domains in pluripotent stem cells that then remain stable between cell types (de Wit et al., 2013). Reproducible spatial associations between fragile loci may be reflected in translocation hot-spots in somatic cells (Roix et al., 2003). For example, Burkitt’s Lymphoma is often characterized by a translocation involving the IgH locus, located on chromosome 14, and the Myc gene promoter, located on chromosome 8 (Roix et al., 2003). Critically, the Myc and IgH loci co-localized during transcription within the nucleus of primary B cells (but not other cell types; Osborne et al., 2007; Wang et al., 2009) supporting the idea that inter-chromosomal interactions can promote disease-associated translocations. Overall, it is evident that genome organization has many common factors across cell type, but is unlikely to be a singular structure. Rather, the spatial organization is dynamic over time and space, helping to regulate the cell’s current needs through cycles of aging, type (function), and current needs (cell-cycle dependent).

## DOES THE SPATIAL ORGANIZATION OF A GENOME ALTER WITH AGE?

DNA damage repair, histone modifications, and chromatin remodeling are all highly affected by the aging process (Burgess et al., 2012). Moreover, cellular aging has been shown to affect the shape of the nucleus and nuclear lamina organization in cell cultures of cells from Hutchinson–Gilford progeria syndrome patients (Bridger and Kill, 2004). The aging process also plays a great role in changes in gene expression, a process shown across species. (Zhan et al., 2007; Brink et al., 2009; de Magalhaes et al., 2009; Park, 2011). Epigenomics is generally considered to be highly dependent on the interplay between the spatial organization of chromosomes and nuclear functions. Therefore, it is reasonable to hypothesize that aging-associated alterations to short- and long-distance regulatory interactions within the spatial organization of the genome affect the development-related expression of critical genes.

It has been shown that in some circumstances similar genes come together in 3D space, regardless of the age of the individual (Gandhi et al., 2006). However, this result is controversial due to its low resolution and observations of the developmental-dependent regulated formation of long-distance interactions associated with the expression of fetal and adult hemoglobin (Tolhuis et al., 2002; Bank, 2006). Specifically, the erythroid-specific genes Hbb-b1 and Eraf, separated by over 20 Mb on the same chromosome, co-localize into a transcriptional factory when actively transcribed (Osborne et al., 2004). Similarly, mice Hbb has been shown to form transcription-associated preferential inter-chromosomal connections with 359 erythroid genes from different genomic locations (Schoenfelder et al., 2010).

## IS IT IMPORTANT TO INCORPORATE THE CHROMOSOME INTERACTION NETWORK INTO FUTURE GWAS/SNP STUDIES?

Extensive efforts have been made to catalog human variation. The most recent versions of dbSNP and the human gene mutation database contain 38,072,522 validated variants (Sherry et al., 2001) and ~100,000 mutations in nuclear genes (Stenson et al., 2009) that are associated with complex human traits, respectively. However, the associations between common-variants (SNPs) and phenotypic traits or diseases held in these databases, and others like them, only describe a small fraction of the overall heritability of complex disease traits (Frazer et al., 2009). Thus, our ability to elucidate functional pathways related to these SNPs has been limited. Part of the reason for missing heritability has been proposed to be bias toward results focused on the coding regions, which comprise only 1.5% of the genome (Consortium et al., 2007). This ignores the rest of the ~2.5–15% of the genome that is estimated to be functionally constrained, yet outside of coding regions (Vernot et al., 2012). Thus, given the fact that enhancers can be located in gene deserts (Harismendy et al., 2011) and can control multiple genes through physical interactions, it is important to determine if SNPs located outside of genes contribute to disease phenotypes through alterations to spatial regulatory interactions.

One caveat to the study of SNPs within non-genic regions is that, while it is known that common SNPs explain a substantial portion of heritability, not all SNPs contribute equally to the heritability of a trait. SNPs in genes explain the most heritability, while those near genes (or in areas regulating them) explain some, and those in non-genic regions (SNP deserts) explain little of the heritability (Smith et al., 2011; Yang et al., 2011; Schork et al., 2013). Despite this, it remains possible that SNPs located outside of coding regions represent a new class of regulatory SNPs that make an important contribution toward explaining heritability.

## ARE THERE ANY CURRENT METHODS FOR ASSOCIATING SNPs AND 3D FUNCTION?

Methods that try to explain the roles of these SNPs in the context of 3D structure have recently begun to be developed. For example, a recently developed database provides functional annotations of SNPs using actual long-range interaction datasets (Wang et al., 2012; Li et al., 2013). By going beyond conservation information and incorporating information from multiple different sources (e.g., HapMap, ENCODE), the GWAS3D database has branded itself as an “efficient solution to interpret the regulatory role of genetic variation in the non-coding regions,” associating SNPs with 3D structure changes. This database brings 3D structure out of LD blocks, but it does not provide a mechanism through which gene deserts would have a functional role within the cell.

## EXAMPLES OF GWAS HITS IN GENE DESERTS PLAYING A FUNCTIONAL ROLE THROUGH 3D INTERACTIONS

Recent advances in the theoretical and experimental methods used to study DNA packaging within cells make it possible to elucidate the biological function and pathways to which SNPs located within gene deserts can contribute. This has been shown in a number of gene deserts, most notably: SNPs within a 1.2 Mb region on chromosome 8q24, a known gene desert, have been implicated in cancer-type-specific interactions with Myc, a highly potent cancer gene > 300 kb away (Amundadottir et al., 2006; Ghoussaini et al., 2008; Ahmadiyeh et al., 2010; Wasserman et al., 2010). This same region has also been implicated in pediatric asthma in an Asian population (Noguchi et al., 2011) and non-syndromic cleft lip in pediatric patients (Grant et al., 2009).

In examples reflecting the complex nature of the metabolic syndrome, several studies have found long-range interactions that regulate metabolic disease pathways. 3D genome interactions within the 9p21 locus were shown to play a functional role for GWAS-significant SNPs associated with coronary artery disease and type 2 diabetes; enhancers in this region with significant STAT-1 binding have the ability to impair interferon-γ signaling response (Harismendy et al., 2011). Li et al. (2013) using the GWAS3D database, showed that SNPs associated with diabetes mellitus have a number of cell-type-specific interactions across the genome, specifically the 1p13 region. This group also explored the regulation of IRS1, active in type 2 diabetes and coronary artery disease, discussing the possible regulatory role of GWAS SNPs in sites 600 Kb and 1 Mb downstream from the IRS1 gene promoter.

Beyond cancer and metabolic syndromes, long-range interactions can have a role in human development. A study on the transcription of Sonic hedgehog (SHH), an important regulator of human development, has shown that its expression can be altered by a SNP in an intron in a gene 1Mb away which acts on a *cis*-acting regulator of SHH (2° of separation; Lettice et al., 2002). This interaction plays a role in the development of preaxial polydactyly.

## CONCLUSION

Since the GWAS era began over a decade ago, the understanding of the genetics of human disease has undergone many significant breakthroughs. Despite this, the likelihood that individual SNPs explain any single heritable disease predisposition decreases with the complexity of the cellular pathways and processes that ultimately contribute to the disease. The “low hanging fruit” from the common-disease, common-variant hypothesis is likely gone, prompting the drive to find new breakthroughs and move the pursuit beyond simply cataloging the deleterious SNPs within genes (Li et al., 2013).

Alterations to the genome can occur to the actual DNA sequence and also at the level of the spatial organization. Individually, or collectively, these changes contribute to diseases in a number of different ways. Therefore, while intergenic SNPs do not affect the coding sequence of any known genes, it remains likely that they contribute to disease phenotypes by changing the 3-dimensional organization of the genome.

While the functional characterization of intergenic SNPs is no easy feat, it is critical that new research identifies and functionally characterizes all variants and variant combinations that make a significant contribution to disease etiology. Expressed quantitative trait loci (eQTL) studies have attempted to link SNPs, changes in gene expression, and phenotype. However, the mechanism by which non-coding SNPs affect expression remains unclear, particularly for trans-eQTLs. Integrating spatial organization, eQTL, and SNP data may provide evidence for direct linkages that explain some of these correlations.

Integrating clinical, computational and molecular approaches to identify changes in pathways mediated by spatial associations with intergenic SNPs will open up avenues of exploration that cannot be otherwise elucidated, defining complex disease in a much clearer manner, and ushering in a new era of disease pathway exploration.

## ETHICAL STANDARDS

All experiments comply with the current laws of the country in which they were performed.

## AUTHOR CONTRIBUTIONS

William Schierding primary author; Wayne S. Cutfield and Justin M. O’Sullivan project leaders (all authors contributed significantly to the writing of this paper).

## Conflict of Interest Statement

The authors declare that the research was conducted in the absence of any commercial or financial relationships that could be construed as a potential conflict of interest.
